# Human Vascular Endothelial Growth Factor A_165_ Expression Induces the Mouse Model of Neovascular Age-Related Macular Degeneration

**DOI:** 10.3390/genes9090438

**Published:** 2018-08-31

**Authors:** Emmi Kokki, Tommi Karttunen, Venla Olsson, Kati Kinnunen, Seppo Ylä-Herttuala

**Affiliations:** 1 A.I. Virtanen Institute for Molecular Sciences, University of Eastern Finland, 70150 Kuopio, Finland; emmi.kokki@uef.fi (E.K.); venla.olsson@uef.fi (V.O); 2 Department of Ophthalmology, Kuopio University Hospital, 70210 Kuopio, Finland; tommi.karttunen@kuh.fi; 3 Department of Ophthalmology, Institute of Clinical Medicine, University of Eastern Finland, 70029 Kuopio, Finland; kati.kinnunen@kuh.fi; 4 Heart Center and Gene Therapy Unit, Kuopio University Hospital, 70029 Kuopio, Finland

**Keywords:** adenovirus, age-related macular degeneration, animal model, gene therapy, gene transfer, neovascularization, subretinal, vascular endothelial growth factor

## Abstract

Vascular endothelial growth factor (VEGF) expression induces age-related macular degeneration (AMD), which is a common vision-threatening disease due to choroidal neovascularization and a fibrovascular membrane. We describe a mouse model of neovascular AMD with the local expression of human VEGF-A_165_ in the eye. We use a transgenic mouse in which human VEGF-A_165_ has been silenced with the loxP-STOP fragment. The choroidal neovascularization and human VEGF-A_165_ expression in the mouse are induced by subretinal adenoviral *Cre* gene delivery. *Cre* gene transfer is compared with adenoviral *LacZ* gene transfer control. We characterize the AMD phenotype and changes in the vasculature by using fluorescein angiography, optical coherence tomography, and immunohistochemistry. At early time points, mice exhibit increases in retinal thickness (348 ± 114 µm vs. 231 ± 32 µm) and choroidal neovascularization area (12000 ± 15174 µm^2^ vs. 2169 ± 3495 µm^2^) compared with the control. At later time points, choroidal neovascularization develops into subretinal fibrovascular membrane. Human VEGF-A_165_ expression lasts several weeks. In conclusion, the retinas display vascular abnormalities consistent with choroidal neovascularization. Together with immunohistochemical findings, these changes resemble clinical AMD-like ocular pathologies. We conclude that this mouse model of *Cre*-induced choroidal neovascularization is useful for mimicking the pathogenesis of AMD, studying the effects of human VEGF-A_165_ in the retina, and evaluating anti-VEGF treatments for choroidal neovascularization.

## 1. Introduction

We hypothesized that overexpression of human vascular endothelial growth factor (VEGF) A_165_ in the eye, using adenoviral gene transfer techniques, could be used to generate a mouse model for choroidal neovascularization. Choroidal neovascularization is a key feature in age-related macular degeneration (AMD), which is a primary cause of vision loss in developed countries [1].

Choroidal neovascularization arising from choroidal vessels originates with the break or defect of Bruch’s membrane as a result of a trauma, a degenerative process, tissue traction, and/or inflammation [2]. In AMD, neovessels arise from the choriocapillaris and invade into the subretinal space through Bruch’s membrane [2]. AMD is also often associated with extracellular deposits, lipofuscin accumulation, geographic atrophy of the retinal pigment epithelium (RPE) and photoreceptors, fibrous scarring, and detachment of the RPE or retina as a result of blood accumulation [3].

VEGF is a potent angiogenic growth factor [4] and its relevance in subretinal and choroidal neovascularization has been well-established in animal models and humans [5]. Expression of VEGF results in leaking neovascularization and vitreous hemorrhages, retinal detachment, and even blindness [6]. Results showed elevated levels of VEGF in aqueous humor of patients [7,8], as well as in different ocular cells, such as RPE [9], the outer nuclear layer [9], and choroidal neovascular [10,11] and fibrovascular membranes [12]. As VEGF plays a critical role in both ocular neovascularization, neutralizing VEGF is the first line of therapy [13]. The development of new treatments relies on animal models that resemble the pathogenesis of human retinal proliferative diseases and allow the study of long-term therapeutic effects.

Existing animal models attempting to simulate AMD do not fully replicate the complex clinical, histological, and angiographic features of the human disease [14]. Preclinical models used today fail to mimic the course of human AMD from CNV to disciform scarring and they usually comprise only a few essential findings of the disease. To address the problem, we created a mouse model for choroidal neovascularization with common clinical AMD findings. To develop this model, we chose to use *Cre* gene transfer into an inducible transgenic mouse line, in which the expression of human VEGF-A_165_ expression is inducible any time point [15]. This enables the use of old mice that mimics the situation in AMD patients, as age is the strongest known risk factor [16]. The eye is anatomically restricted and divided into compartments, which allow the precise and targeted delivery of gene therapy [17]. As subretinal adenoviral injection more efficiently transduces RPE cells than intravitreal injection [18,19,20,21], it is a natural choice as a delivery route for choroidal neovascularization models. Our model with local human VEGF-A_165_ expression in the eye enables studies of the pathogenetic mechanisms and new therapeutic approaches to the treatment of human VEGF-A_165_ overexpression and neovascularization in the eyes.

## 2. Materials and Methods

Adult female and male transgenic mice (*n* = 44) with a loxP-STOP fragment inactivated hVEGF-A_165_ expression cassette [15] were used for the study. Mice were housed in regular 12-hr light/dark cycle and food and water were available ad libitum. All animal procedures were approved by the Animal Experiment Board in Finland (license ESAVI-2016-000851) and carried out according to the guidelines of the Experimental Animal Committee of the University of Eastern Finland and in accordance with the European Communities Council Directive 2010/63/EU. The research complied with the commonly-accepted “three Rs”: Replacement, reduction, and refinement of experimental animals.

For the procedures, mice were anesthetized subcutaneously with ketamine (2-(2-chlorophenyl)-2-(methylamino)cyclohexan-1-one) (Ketaminol vet 50 mg/mL, Intervet, Boxmeer, The Netherlands) and medetomidine (5-[1-(2,3-dimethylphenyl)ethyl]-1H-imidazole;hydrochloride) (Domitor vet 1 mg/mL, Orion Pharma, Espoo, Finland). Atipamezole (5-(2-ethyl-1,3-dihydroinden-2-yl)-1H-imidazole;hydrochloride) (Antisedan vet 5 mg/mL, Orion Pharma, Espoo, Finland) was used as reversal of the sedatives. Phenylephrine (3-[(1R)-1-hydroxy-2-(methylamino)ethyl]phenol) (Oftan Metaoksedrin 100 mg/mL, Santen Oy, Tampere, Finland) and tropicamide (N-ethyl-3-hydroxy-2-phenyl-N-(pyridin-4-ylmethyl)propanamide) (Oftan Tropicamid 5 mg/mL, Santen Oy, Tampere, Finland) were used to induce mydriasis, and oxybuprocaine (2-(diethylamino)ethyl 4-amino-3-butoxybenzoate) eye drops (Oftan Obucain 4 mg/mL, Santen Oy, Tampere, Finland) as analgesic. Carbomer (2-methylbutanoic acid) eye gel (Viscotears 2 mg/mL, Bausch & Lomb Nordic AB, Berlin, Germany) was used as a lubricant. Animals were divided into two groups. One group of mice was injected subretinally with recombinant E1-partial E3-deleted first generation serotype five adenovirus *Cre* (3.5 × 10^10^ pfu/mL) and another group with adenoviral *LacZ* (1.8 × 10^10^ pfu/mL) control. 2 µL of virus was injected subretinally using a Hamilton syringe and a 33-gauge needle. Injection was performed transsclerally to one eye. Eyes were visualized under a microscope and mice without a bleb or with bleeding after the injection were withdrawn from the study. Animals were sacrificed 2, 6, or 12 weeks after the subretinal injection using carbon dioxide. The number of mice and eyes studied per group included the following: week 2 Cre (*n* = 8) and LacZ (*n* = 9), week 6 Cre (*n* = 6) and LacZ (*n* = 6), and week 12 Cre (*n* = 8) and LacZ (*n* = 7).

The peripapillar area of the retina was examined with Heidelberg Spectral domain optical coherence tomography (OCT) and fluorescein angiography (FA) a day before subretinal injection and a day before the sacrifice (*n* = 41). Three mice were excluded because of cataract or other reason that prevented imaging the retina. The images were analysed with Heidelberg Eye Explorer version 1.9.10.0 (Franklin, MA, USA). The custom-made surface was created for anesthetized mice and optical coherence tomography images were obtained with a 30° lens. Standard human settings in the program were used altering the focus up to +40 D. For fluorescein angiography, 0.2 mL of 2% fluorescein (3′,6′-dihydroxyspiro[2-benzofuran-3,9′-xanthene]-1-one) (Fluorescite 100 mg/mL, Novartis Finland Oy, Espoo, Finland) was injected intraperitoneally and a wide angle 55° lens was used. Images were obtained 2–3 min after the injection. Furthermore, the images were obtained from the oedematous areas and peripheral retina where changes were seen in comparison with baseline images. Retinal thickness in the peripapillar area was automatically calculated from a 1-mm diameter circle.

The following day of the final OCT and FA imaging, mice were sacrificed and tissues were fixed in 4% paraformaldehyde overnight, embedded in paraffin, and sectioned at 4 µm thickness. Hematoxylin-eosin, PicroSirius Red (ab150681, Abcam, Cambridge, UK), and terminal deoxynucleotidyl transferase dUTP nick-end labeling (TUNEL) (TACS 2 TdT-Fluor in situ apoptosis detection kit, Trevigen, Gaithersburg, MD, USA) stainings were performed. For immunostainings, the following antibodies were used: CD34 (MEC14.7, Hycult Biotech, Uden, The Netherlands), glial fibrillary acidic protein (GFAP) (Z0334, Dako, Santa Clara, CA, USA), F4/80 (MCA497R, Bio-Rad, Hercules, CA, USA), β-gal (AB1211, EMD Millipore, Billerica, MA, USA), and VEGF (ab52917, Abcam). Secondary antibodies with Alexa Fluor 488 and 594 conjugates (ThermoFischer Scientific, Waltham, MA, USA) were used for detection. Mounting medium with nuclear counterstain 4′,6-diamidino-2-phenylindole (DAPI) (H-1200, Vector Laboratories, Burlingame, CA, USA) was used with fluorescent secondary antibodies. For detection of VEGF, a peroxidase staining kit (ABC Vectastain Elite, PK-6100, Vector Laboratories) and peroxidase substrate (VIP Substrate, SK-4600, Vector Laboratories) were used. Methyl Green (H-3402, Vector Laboratories) was used as a nuclear counterstain. Fluorescence imaging using emission wavelength of 470 nm was used to study autofluorescence in the sections. Sudan Black B (199664, Merck, Darmstadt, Germany) was used to diminish autofluorescence in other retinal sections as previously described [22]. Photographs were taken with a Nikon Eclipse Ni electron microscope and Nikon DS-Ri2 and DS-Qi2 cameras (Tokyo, Japan). NIS-Elements AR. version 4.50.00 was used to analyse photographs. Adobe Photoshop CS5 (San Jose, CA, USA) was used to generate the merged images of different fluorescent channels. The maximum number of apoptotic and CD34 positive areas were calculated from a single image taken with a 10× objective. The maximum fibrotic scarring area and intensity of Sirius Red staining were calculated from a single image taken with 4× objective.

Liver samples and whole eyes were used for human VEGF-A_165_ messenger RNA (mRNA) analysis using real-time quantitative polymerase chain reaction (RT-qPCR). To study the mRNA expression in the eyes, additional mice were subretinally injected at two weeks (*n* = 3) and six weeks (*n* = 4). Eyes at the same time point were pooled together and RNA was isolated immediately after scarification. Liver samples were snap frozen in liquid nitrogen before RNA extraction. Total RNA was isolated with RNeasy^®^ Mini Kit (74104, Qiagen, Hilden, Germany) and DNAse treated with TURBO DNA-free Kit (AM1907, ThermoFischer Scientific). Complementary DNA (cDNA) was synthesized using random hexamer primers (SO142, ThermoFischer Scientific) and Revert Aid Reverse Transcriptase (EP0441, ThermoFischer Scientific). Gene expression was determined with quantitative PCR (StepOne Plus instrument and software version 2.2.2, Applied Biosystems, Foster City, CA, USA) using a probe based on inner primers for hVEGF-A (Hs00900055_m1, Applied Biosystems) and cyclophilin A (PPIA) (Mm03302254_g1) for the eyes and ribosomal protein lateral stalk subunit P0 (Rplp0) (Mm.PT.58.4389402, Integrated DNA Technologies, Skokie, IL, USA) for the livers. Enzyme-linked immunoassay assay (ELISA) was carried out to quantify human VEGF-A_165_ protein in plasma samples and in liver and lung homogenate (human VEGF Quantikine ELISA Kit, DVE00, R&D Systems, Minneapolis, MN, USA). Circulating hVEGF-A_165_ was measured from heart puncture plasma samples at the time of sacrifice. Homogenization buffer (T-PER [Tissue Protein Extraction Reagent], ThermoFisher Scientific) and protease inhibitors (cOmplete, Mini, ethylenediaminetetraacetic acid (EDTA) -free Protease Inhibitor Cocktail, Merck) were added to tissue samples before homogenization. The total protein concentration of the tissue was measured with the bicinchoninic acid (BCA) Protein Assay (23225, ThermoFischer Scientific) before ELISA analyses.

Two-way analysis of variance (ANOVA) and Bonferroni post-test (GraphPad Prism, v. 5.03) were used for the statistical analysis of apoptotic cell assay, Sirius Red staining, CD34-positive area, and qPCR results. A value of *p* ≤ 0.05 was considered significant.

## 3. Results

The ocular effects and biodistribution after adenoviral vector-mediated *Cre* and *LacZ* control gene transfer were studied 2, 6, and 12 weeks after the subretinal injection. The optical coherence tomography and fluorescein angiography were used to compare the retinal thickness and fluorescence with intact non-injected eyes. Several stainings were used to study the AMD-like findings and inflammation in the eye after gene transfer. Biodistribution of *Cre*-induced human VEGF-A_165_ expression outside the eye was studied at all three time points. To study human VEGF-A_165_ expression in the eye, seven additional mice were injected and studied two and six weeks after gene transfer.

### 3.1. Changes in the Retinal Thickness and Vascularization

**Figure 1:**
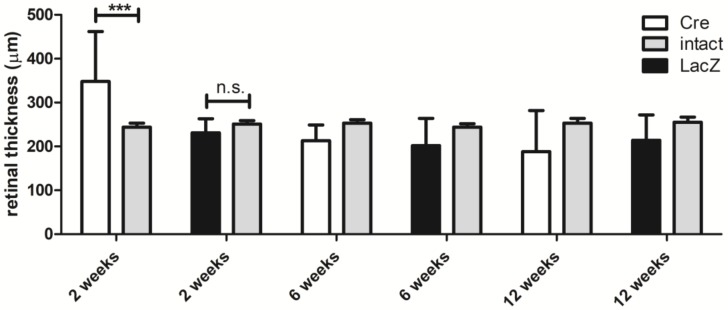
Mean retinal thickness measured by optical coherence tomography at different time points compared with the intact eye of the same animal measured before the injection. Results are presented as mean ± standard deviation (SD). *** *p* ≤ 0.001.

Figure 1 shows retinal thickness at different time points. The optical coherence tomography images were used to study the retinal thickness in intact eyes before the injection and after *Cre* and *LacZ* injections at different time points. To compare the retinal thickness of intact non-injected eyes versus subretinally injected eyes, the mean retinal thickness at the baseline was measured (251 ± 10 µm) in each mouse (*n* = 41) at each time point before the injection. After two weeks, Cre mice had significantly thicker retina (348 ± 114 µm) than LacZ mice (231 ± 32 µm). At later time points, both groups tended to display retinal atrophy and decreasing retinal thickness, but it was not statistically significant in comparison with the non-injected eyes. 

**Figure 2:**
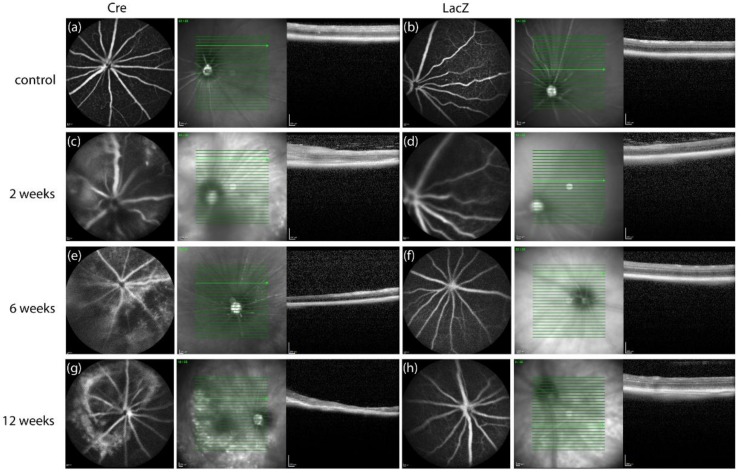
Fluorescein angiography (FA) and optical coherence tomography (OCT) images taken before gene transfer and after *Cre* and *LacZ* injections: (**a**,**b**) normal OCT and FA before injection, (**d**,**f**,**h**) normal OCT and FA after *LacZ* injection. (**c**) Diffuse hyperfluorescence seen in FA and thicker retina in OCT two weeks after *Cre* injection. (**e**) Hyperfluorescent and hypofluorescent areas in FA and thin retina in OCT six weeks after *Cre* gene transfer. (**g**) Hyperfluorescent circular area with hypofluoresence in the middle of FA image and thin and atrophic retina seen in OCT 12 weeks post-*Cre* injection.

Morphological changes were observed in optical coherence tomography images in both groups (Figure 2). Cre mice developed disoriented and swollen retinal layers around the injection site and subretinal fluid was seen. In LacZ groups, the disorientation of retinal layers was also seen but to a lesser extent compared with Cre groups. The hyperfluorescence was seen in both groups after injections. Similarly, in the optical coherence tomography images, the effect was the strongest at two weeks (Figure 2c,d) and diminished at later time points. Atrophic areas were also seen as hypofluorescent areas in the retina. After two weeks, the hyperfluorescence diminished and atrophic findings emerged (Figure 2e,g). Hypofluorescent areas were seen in the thin atrophic retinas.

The fluorescein angiography images were analyzed by rating the hyperfluorescence seen in the images (Table 1). The findings in Stage 1 were normal and similar to FA findings before injections. In Stage 2, there was hyperfluorescence in a small area, and in Stage 3, there was hyperfluorescence in a large area or diffuse hyperfluorescence was observed. In Stage 4, there were tortuous vasculature and diffuse hyperfluorescence. Two weeks post-*Cre* injection, in three-eighths (37.5%) of the eyes, hyperfluorescence was rated as normal or minimally increased. In five-eighths (62.5%) of the eyes, diffuse hyperfluorescence and tortuous vasculature was seen and the retina was remarkably thicker. In the control group, there was also some hyperfluorescence (Stages 2–3) in three-eighths (37.5%) of the eyes, but in these eyes, retinal thickness was lower compared with *Cre*-injected eyes. OCT and FA findings were also compared to CD34 positive area. For two mice, CD34 staining was not available.

**Figure 3:**
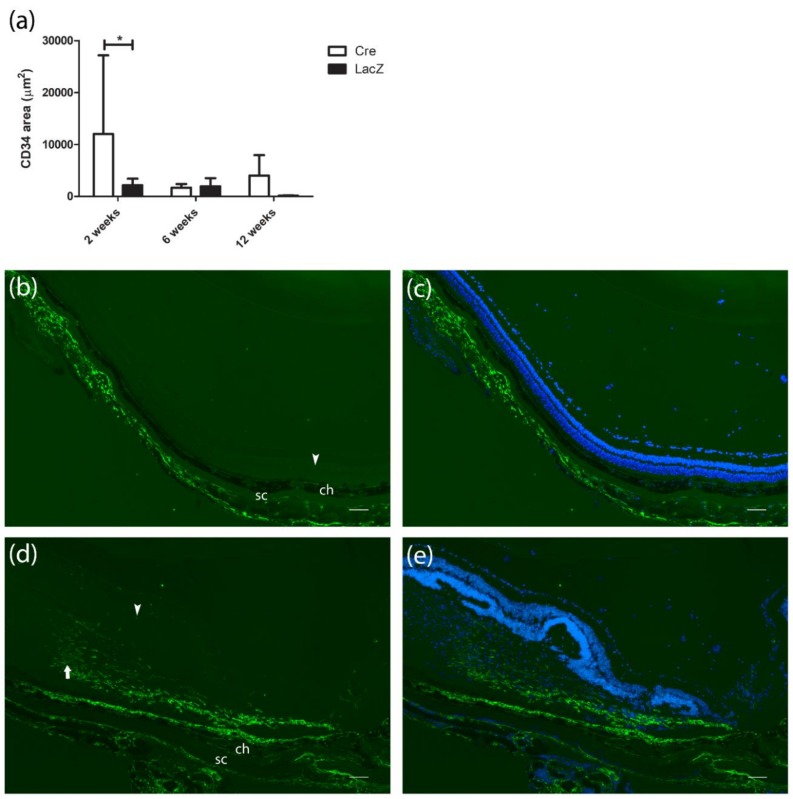
Comparison of CD34 positive endothelial cells (green) in *Cre-* and *LacZ*-injected mice with nuclear counterstain 4′,6-diamidino-2-phenylindole DAPI (blue). (**a**) CD34 positive area in the retina and subretinal membranes. Results are presented as mean ± SD. * *p* ≤ 0.05. (**b**,**c**) Few normal deep retinal vessels (arrowhead) in the retina two weeks after *LacZ* gene transfer. (**d,e**) Single retinal endothelial cells and massive subretinal neovascular membrane (arrow) under the swollen retina two weeks after *Cre* injection. Scale bar is 100 µm. ch: Choroid, sc: Sclera.

CD34 staining was used to visualize the vascular endothelium. The area of CD34 positive cells was larger in the *Cre*-injected group (12,000 ± 15,174 µm^2^) compared with the *LacZ*-injected group (2169 ± 3495 µm^2^) at the two-week time point (Figure 3a). Both groups had a few CD34 positive cells in the retina. In addition, *Cre* injection resulted in a massive subretinal neovascular membrane two weeks after gene transfer (Figure 3d,e). The most extensive CD34 positive vascularization correlated with the retinal thickness and hyperfluorescence (Table 1).

### 3.2. Histological Findings Related to Age-Related Macular Degeneration

**Figure 4:**
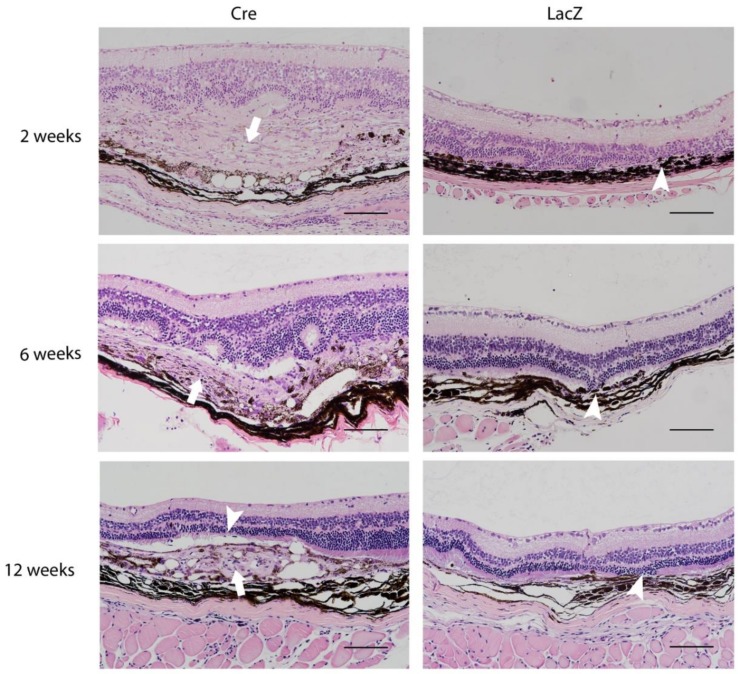
The morphological retinal changes including neovascularization (arrow) and thinning of retinal layers (arrowhead) of *Cre-* and *LacZ*-injected eyes. Changes were most prominent two weeks after *Cre* injection and evolved toward proliferative age-related macular degeneration at later time points. Morphological changes were found to a lesser extent in LacZ groups. Scale bar is 100 µm.

Morphological changes in the *Cre*-injected group were most prominent two weeks after the subretinal injections. Morphological changes were found comprehensively two weeks post-*Cre* injection, whereas at the later time points, changes were local around the site of the injection. The major findings for *Cre*-injected eyes are summarized in Table 2. These findings included changes such as neovascular membrane (Figure 4, arrow). The increased retinal thickness due to subretinal swelling, seen in optical coherence tomography imaging, was also visible in hematoxylin-eosin stainings. The thinning of photoreceptor layer or outer nuclear layer (arrowhead) was the most common finding in the LacZ control group at the site of the injection two weeks after gene transfer. Some thinning of the outer retinal layers was seen also in *Cre*-injected eyes (arrowhead). The contralateral intact eyes did not show any changes.

**Figure 5:**
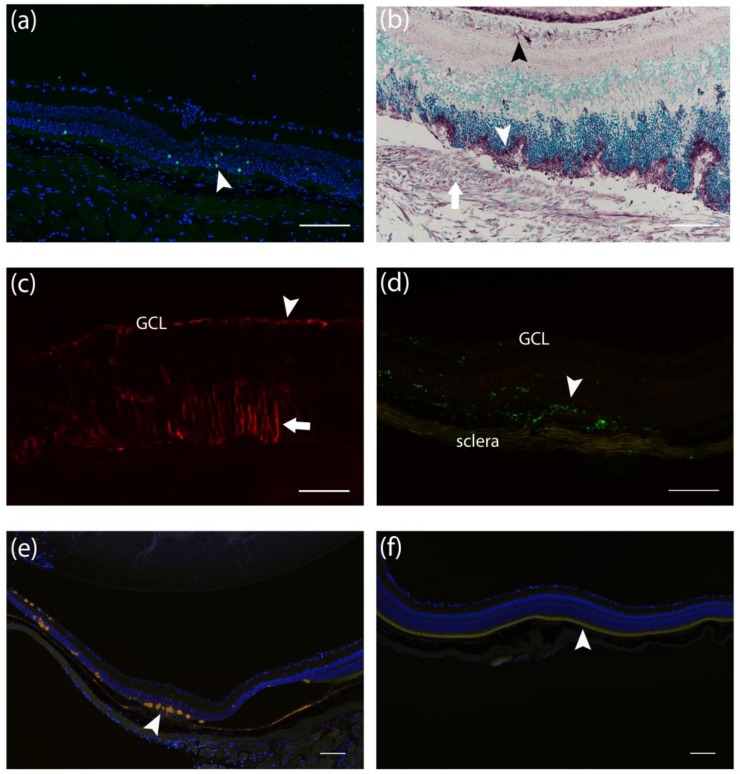
Morphologic changes and transgene expression in the *Cre* and *LacZ* injected eyes. (**a**) β-gal expression (arrowhead) after *LacZ* injection was seen two weeks after gene transfer but not at later time points. (**b**) VEGF-A expression (violet) in ganglion cell layer (black arrowhead), photoreceptors (arrowhead), and neovascular membrane (arrow) in the eye of *Cre*-injected mouse. (**c**) Glial fibrillary acidic protein (GFAP) immunoreactivity was observed in the nerve fiber layer (arrowhead) and Müller cells (arrow) in the outer retina post-*Cre* injection. (**d**) In the Cre group, F4/80 positive macrophages were seen in the retina and subretinal layers. (**e**,**f**) Retinal autofluorescence (yellow) with DAPI nuclear counterstain (blue). In *Cre*-injected retina (e), drusen-like lipofuscin deposits (arrowhead) and the loss of photoreceptors were seen. Intact photoreceptor layer (f, arrowhead) was observed in *LacZ*-injected eyes. Scale bar is 100 µm. GCL: Ganglion cell layer.

β-gal staining showed *LacZ* positive cells two weeks after the subretinal injections. Transgene expression was found mostly in the outer nuclear layer (Figure 5a). Anti-human/mouse VEGF-A antibody showed positivity in photoreceptor cells and in the ganglion cell layer in both Cre and LacZ groups, but also in the neovascular and fibrovascular membrane in the Cre group (Figure 5b). GFAP expression was seen in astrocytes in the nerve fiber layer in all injected eyes. Additional extensive GFAP immunoreactivity was found in Müller cells in the outer retina in *Cre*-injected mice (Figure 5c). Müller cell activation was found around the neovascular membranes but not at the direct sites of the injection. Some *LacZ*-injected retinas also showed minor glial cell activation two and six weeks post-injection. F4/80 positive macrophages were seen mostly in *Cre*-injected mice, but also to a lesser extent in the LacZ group. F4/80 staining showed an increased number of macrophages in all retinal layers and in subretinal neovascular membranes (Figure 5d). F4/80 positive cells were also found surrounding autofluorescent deposits and in fibrovascular membranes at later time points. Autofluorescence from drusen-like deposits was seen mostly in the subretinal space at retinal pigment epithelium, subretinal neovascular membrane, and atrophic outer nuclear layer, and occasionally in the ganglion cell layer (Figure 5e). In the *Cre*-injected group, the loss of photoreceptors was also seen, whereas in the LacZ group, the layer was intact in most eyes at the 6- and 12-week time points (Figure 5f).

### 3.3. Presence of Apoptotic Cells

**Figure 6:**
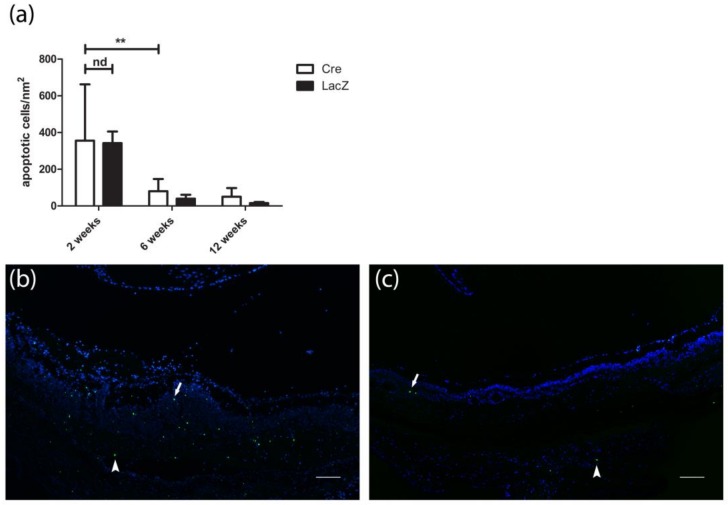
Apoptosis in the retina after subretinal injection in terminal deoxynucleotidyl transferase dUTP nick end labeling (TUNEL) staining (green) with DAPI nuclear counterstain (blue). (**a**) No statistical difference in the number of apoptotic cells was detected between LacZ and Cre groups at the site of the injection at any of the time points, but the number of apoptotic cells decreased statistically during time. Results are presented as mean ± SD. ** *P* ≤0.01. (**b**) Apoptotic cells were mostly seen in the subretinal membrane (arrowhead) and in the outer retinal layers (arrow) two weeks after the *Cre* injection. (**c**) Few positive cells were observed in the outer nuclear layer (arrow) and in the subretinal layers (arrowhead) 12 weeks after the subretinal *Cre* injection. Scale bar is 100 µm.

Terminal deoxynucleotidyl transferase dUTP nick-end labeling (TUNEL) showed apoptosis in the outer retinal layers in both groups at the site of subretinal injections (Figure 6a) without statistical difference between the groups at any time point. The number of apoptotic cells was most extensive two weeks after the subretinal injections in the *Cre*-injected group (307 ± 356 cells/mm^2^) and in the *LacZ*-injected group (342 ± 190 cells/mm^2^). Apoptosis decreased significantly at the six-week time point in both groups (80 ± 67 cells/mm^2^ and 39 ± 53 cells/mm^2^, respectively) and only a few positive cells were seen two weeks after injection (Figure 6c).

### 3.4. Development of Fibrovascular Membrane

**Figure 7:**
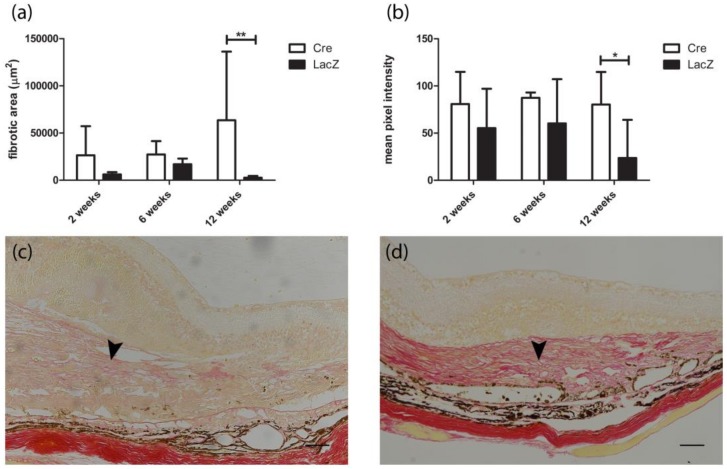
Fibrotic scarring in Sirius Red stained samples. (**a**,**b**) Subretinal collagen formation was seen at all time points in the *Cre*-injected group. Twelve weeks after *LacZ* injection, mild positive staining was seen only in two subretinally injected eyes. Intensity of the staining increased at later time points. (**c**) Faint positive collagen staining two weeks after *Cre* injection (arrowhead). (**d**) Subretinal fibrovascular membrane was clearly seen 12 weeks post-*Cre* gene transfer (arrowhead). Results are presented as mean ± SD. * *p* ≤ 0.05, ** *p* ≤ 0.01. Scale bar is 100 µm.

Figure 7 presents the maximum measured area and intensity of the Sirius Red staining of the subretinal fibrotic scar. Twelve weeks after *Cre* injection, the fibrotic area was significantly larger (63,599 ± 72,694 µm^2^) than in the LacZ group (2738 ± 4945 µm^2^). The collagen formation was seen lining the retinal pigment epithelium subretinally. In addition, the staining in *Cre*-injected mice’s eyes was significantly more intensive than at earlier time points (Figure 7b,c).

### 3.5. Expression Levels of Human Vascular Endothelial Growth Factor A_165_ in the Eye and Off-Targets

**Figure 8:**
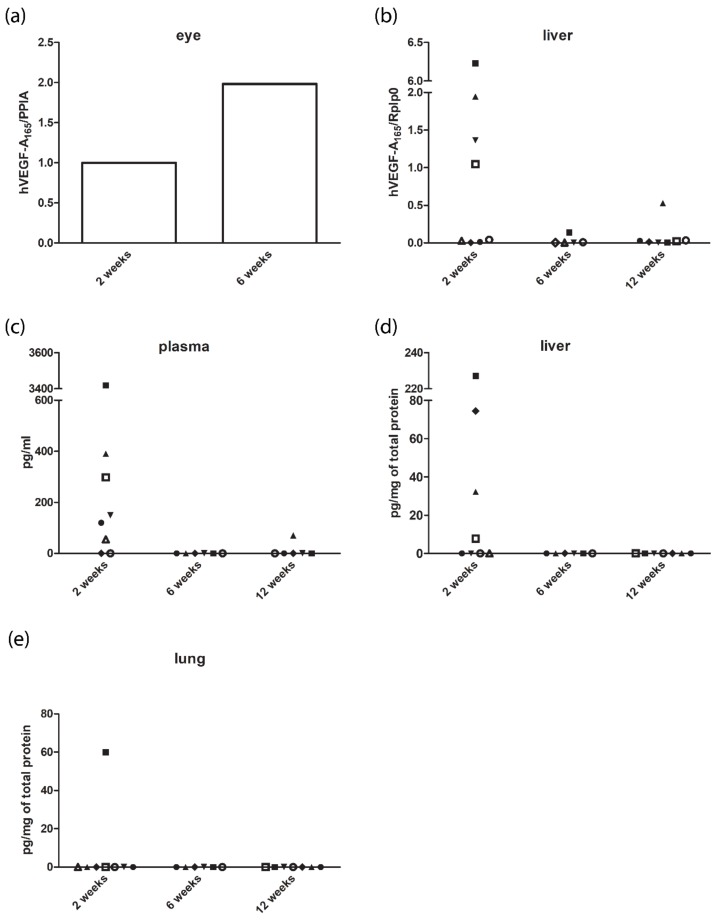
Human VEGF-A_165_ messenger RNA (mRNA) and protein expression in the eye and off-targets. (**a**) Real-time polymerase chain reaction (RT-PCR) analysis of the expression of hVEGF-A_165_ in whole eyes. Cyclophilin A (PPIA) normalized relative mRNA expression of hVEGF-A_165_ was two-fold higher six weeks after *Cre* injection compared with the two-week time point. (**b**) RT-PCR analysis of the expression of hVEGF-A_165_ in liver samples. Ribosomal protein lateral stalk subunit P0 (Rplp0) normalized mRNA expression of hVEGF-A_165_ was the highest two weeks after *Cre* injection. (**c**–**e**) hVEGF-A_165_ protein levels in plasma, liver, and lung homogenate after *Cre* gene transfer, respectively. The same symbols in each graph represent the same animal.

Pooled samples of mice’s eyes showed a two-fold expression in human VEGF-A_165_ mRNA levels six weeks after *Cre* injection compared with the two-week time point (Figure 8a). The mRNA expression of human VEGF-A_165_ was detected in all liver samples of the *Cre*-treated mice (Figure 8b). The highest levels were detected two weeks after gene transfer followed by a low-level expression at later time points. Detectable levels of human VEGF-A_165_ protein in the plasma samples were found in 75% (six-eights) of *Cre*-injected mice two weeks after the injection and in one mouse 12 weeks after the injection (Figure 8c). Tissue homogenate expressed human VEGF-A_165_ only two weeks after *Cre* gene transfer. A total of 50% (four-eights) of liver homogenate (Figure 8d) and one sample of lung homogenate (Figure 8e) showed detectable levels of the protein.

## 4. Discussion

Pathologic conditions affecting ocular blood vessels and causing neovascularization pose direct threat to human vision [5]. The study of new treatments for neovascular ocular diseases highly depends on the development of reproducible and reliable pre-clinical models. Although animal models have already been developed to study the pathobiology of ocular angiogenesis, the course of progression and the duration of symptoms, as well as the molecular and cellular basis of the disease, vary from clinical perspective. To provide an alternative solution to existing models, we reported here a novel mouse model of AMD and choroidal neovascularization expressing human VEGF-A_165_ with additional AMD-like features, such as the presence of macrophages and break in Bruch’s membrane.

Damage to Bruch’s membrane, VEGF expression, or inflammatory cytokines are commonly used key features in the generation of animal models of choroidal neovascularization [23]. The most commonly-used methods to develop animal models of choroidal neovascularization include laser or surgical induction or the use of transgenic mice. Choroidal neovascularization models based on the overexpression of VEGF are created either by administration of VEGF into animals or by using transgenic mice that express VEGF. Nevertheless, the models studied to date indicate that VEGF overexpression alone is insufficient for developing choroidal neovascularization [23]. Our mouse model combines VEGF overexpression with the subretinal needle puncture-caused break in Bruch’s membrane, which leads to neovascularization. One of the other models successfully developing choroidal neovascularization after subretinal injection is the AAV.shRNA.sFLT-1 model, in which a short hairpin RNA targets VEGF receptor-1 [24,25].

The other common types of animal models of choroidal neovascularization are based on laser or mechanically-induced breaks in Bruch’s membrane [14,23]. The laser-induced choroidal neovascularization model is one of the most often used as it is relatively rapid to develop [26]. The limitation of laser-induced models is that the choroidal neovascularization disappears approximately a month after lasering [27,28,29]. As the retina is partially burned in laser-induced choroidal neovascularization, anatomic discrepancies exist to a greater degree than is typical for human AMD due to significant damages to the overlying neural retina [14].

Another limitation in many rodent models is that only a small percentage of mice develop choroidal neovascularization and areas of neovascularization remain low [30,31]. Our study presents a novel mouse model in which 75% of the mice developed subretinal neovascular membranes only two weeks after the induction of human VEGF-A_165_ expression. Twelve weeks after the induction, the percentage of mice that developed characteristics of AMD was even higher. In the current work, the presence of choroidal neovascular membrane was demonstrated by HE and immunohistochemical stainings as well as by OCT- and FA-imaging. *Cre*-induced human VEGF-A_165_ expression led to vascular changes in the mouse retina. In the Cre group, the fluorescein angiography image findings correlated with retinal thickness in optical coherence tomography. Also, the CD34-positive subretinal vascular area correlated with the findings in OCT and FA images.

The subretinal vascular area developed into fibrosis during the study. Sirius Red staining showed fibrous subretinal scarring up to 12 weeks after the *Cre* gene transfer. Both the area and the intensity of the collagen staining increased during the study, whereas the number of CD34-positive endothelial cells decreased during the same time, thus indicating the fast development of the model toward the proliferative stage of AMD. Although in humans neovascular AMD develops into a cicatricial stage with disciform scar as a response to wound healing [10,12,32,33,34], only a few rodent models with subretinal fibrosis are available [29] despite the success in inducing choroidal neovascularization [24,25,35]. There are some rodent models developing fibrovascular membranes but only in small areas [36,37]. One of the models of subretinal fibrosis is a murine model utilizing laser photocoagulation and injection of macrophages into the subretinal space [38,39,40]. One major concern is the complexity of the model given its multiple steps, decreasing the reproducibility of the model. Similar to our findings, a mouse model exploiting laser-induced choroidal neovascularization showed a decrease in neovascular membrane and an increase in subretinal fibrosis within weeks [41]. This is consistent with the findings in AMD patients in whom fibrous scarring subsequently develops into hemorrhages caused by subretinal vessel growth [42]. Although choroidal neovascularization in murine models was observed within days after induction [27,41], the development of fibrosis may take several weeks [24,41]. Thus, to follow the progression of the disease, older mice should be studied for longer periods. Animal models with sustained VEGF expression, which allow the study of long-term therapeutic effects, are valuable for pre-clinical treatment trials.

In immunohistochemical staining, we found VEGF protein expression in Müller cells and photoreceptors but also in choroidal neovascularization and fibrovascular membranes. When the whole eye was studied in qPCR, mRNA levels of human VEGF-A_165_ were about two-fold higher six weeks after *Cre* injection, compared with two week post gene transfer. Besides the injected eyes, human VEGF-A_165_ was also found in off-target organs and plasma. Some other studies have also reported transgene expression outside the eye after intraocular gene delivery to rodent eyes [43,44,45,46,47]. Detectable mRNA and protein levels outside the eye indicate that the virus has entered the bloodstream, possibly due to blood-retina-barrier breakdown as a result of the injection. Despite the systemic expression of human VEGF-A_165_, we did not see adverse or angiogenic effects outside the eye. Detectable levels of human VEGF-A_165_ mRNA were found in all livers of *Cre*-injected mice. Expression levels were the highest two weeks after the injection, but only very low levels were detected at the later time points. A previous study on mice with *Cre*-inducible human VEGF-A_165_ expression also showed reduction in protein levels in plasma and tissues only a month after the intravenous adenoviral *Cre* injection [15]. Decrease in human VEGF-A_165_ levels with time could be due to immune responses against adenovirus or the toxicity of *Cre*. Apoptosis of *Cre*-expressing cells could explain the decrease in human VEGF-A_165_ levels as an increased number of apoptotic cells was observed two weeks after the adenoviral injection. Apoptosis was recorded in both groups and the number of apoptotic cells decreased only six weeks after the gene transfer. This indicates that the trauma caused by the injection or adenovirus itself led to apoptosis.

In addition to apoptosis, atrophy in the retina was the most dominant finding observed in *LacZ*-injected eyes, possibly explained by trauma. Two weeks after injection, trauma caused by subretinal injection was still observed, which might explain the CD34-positive area in the LacZ group. This effect was reported previously [48]. Injection itself also explains that a few *LacZ* injected retinas expressed GFAP at early time points, as seen in other models with injection of control adenoviral vectors or saline [49]. In comparison, we detected extensive glial cell activation even 12 weeks post-*Cre* injection within the entire retina, showing the response to retinal injury or photoreceptor degeneration [50]. Loss of photoreceptor layer was detected at some stage in all *Cre*-injected mice at all studied time points. It is indisputable that the needle puncture for gene transfer causes some of the retinal findings seen in our model. Nevertheless, a break in Bruch’s membrane is necessary for choroidal neovascularization to develop [23].

Choroidal neovascularization in AMD has been correlated with the presence of F4/80-positive macrophages [51]. Macrophages have an important role in the pathogenesis of AMD, as they express several cytokines and growth factors, including VEGF [52], thus possibly promoting the growth of choroidal neovascularization. In human eyes with AMD, macrophages are located near pathologic neovascularization, in degrading areas of Bruch’s membrane and in the choroidal neovascular membrane [53]. In mouse models, F4/80-positive cells have been found in the retina, RPE, and choroidal tissue [54,55]. In our study, subretinal administration of Cre resulted in an increase in F4/80-positive macrophages in the retina and subretinal membranes and also hematoxylin-eosin stained sections showed pigmented macrophages in close proximity with the newly developed vessels in the retina. We also detected accumulation of drusen-like lesions of swollen autofluorescent macrophages, which were previously observed in macrophages positive for F4/80 [56] or other macrophage markers [56,57,58]. Studies have reported age-dependent autofluoresence in healthy rodents [58,59] and also in mice with AMD-like genotype [60] or phenotype [56]. Autofluoresence is likely caused by lipofuscin—yellow-brown pigment granules composed of lipid containing residues of lysosomal digestion [58]. Accumulation of lipofuscin into macrophages and microglia may interfere with their ability to clear debris and thus contribute to the pathogenetic mechanisms of many age-related ocular diseases, including age-related AMD [58,61].

Animal models for complex diseases, such as AMD, remain challenging. A suitable model should recapitulate many of the clinical manifestations of the disease, have accessible in vivo imaging, and develop choroidal neovascularization within a reasonable time frame. A persistent model is needed for the longitudinal studies of ocular treatments. We conclude that subretinal *Cre* injection into loxP-STOP-hVEGF-A_165_ transgenic mouse eye creates several features of AMD. The induced mouse model provides a tool for studying the early progression of choroidal neovascularization and later stages of fibrovascular AMD progression. This model provides possibilities for the development of choroidal neovascularization-inhibiting agents.

## Figures and Tables

**Table 1:** Optical coherence tomography measured central retinal thickness (CRT) at baseline compared with CRT, fluorescein angiography (FA) stage of hyperfluorescence, and CD34 positive capillary area (μm^2^) two weeks after injection.

	Baseline			2 Weeks			Baseline			2 Weeks	
Cre	CRT		CRT	FA	CD34 Area	LacZ	CRT		CRT	FA	CD34 Area
mouse 1	241		255	2	106	mouse 9	243		194	1	272
mouse 2	250		585	4	n/a	mouse 10	253		235	2	530
mouse 3	261		380	4	10350	mouse 11	248		185	3	603
mouse 4	250		267	2	1067	mouse 12	254		255	1	37
mouse 5	240		310	4	41797	mouse 13	252		n/a	n/a	n/a
mouse 6	234		301	4	21304	mouse 14	250		205	3	6776
mouse 7	244		256	1	423	mouse 15	270		272	1	171
mouse 8	232		430	4	8952	mouse 16	241		248	1	8741
						mouse 17	253		254	1	220
mean ± SD	244 ± 9.5		348 ± 114	3.1±1.2	12000 ± 15174	mean ± SD	251 ± 8.5		231 ± 32.2	1.6 ± 0.9	2169 ± 3495

Note: The stage of hyperfluorescence: 1—normal; 2—minor hyperfluorescence, 3—major or diffuse hyperfluorescence, 4—vasculature changes and diffuse hyperfluorescence, n/a: Not available.

**Table 2:** Retinal and subretinal changes in *Cre*-injected mice.

Characteristic	2 Weeks	6 Weeks	12 Weeks
Subretinal swelling	++	ND	ND
Macrophage infiltration	++	+	+
Photoreceptor loss	+++	+++	+++
Drusen-like deposit *	ND	+	+
GFAP activation	+++	++	+++
ONL atrophy or loss	++	+++	++
INL atrophy	+	+	+
Infiltrating cells in the vitreous ^¶^	5/8	1/6	1/8

+: One quadrant; ++: Two quadrants; +++: Three quadrants; ++++: Four quadrants; ND: Not detected, GFAP: Glial fibrillary acidic protein, ONL: Outer nuclear layer, INL: Inner nuclear layer, *: Drusen-like deposits detected, ^¶^: The number of mice.
